# The Effect of Feeding Bt MON810 Maize to Pigs for 110 Days on Intestinal Microbiota

**DOI:** 10.1371/journal.pone.0033668

**Published:** 2012-05-04

**Authors:** Stefan G. Buzoianu, Maria C. Walsh, Mary C. Rea, Orla O’Sullivan, Fiona Crispie, Paul D. Cotter, R. Paul Ross, Gillian E. Gardiner, Peadar G. Lawlor

**Affiliations:** 1 Teagasc, Pig Development Department, Animal and Grassland Research and Innovation Centre, Moorepark, Fermoy, Ireland; 2 Department of Chemical and Life Sciences, Waterford Institute of Technology, Waterford, Ireland; 3 Teagasc, Food Research Centre, Moorepark, Fermoy, Ireland; 4 Alimentary Pharmabiotic Centre, University College Cork, Cork, Ireland; Argonne National Laboratory, United States of America

## Abstract

**Objective:**

To assess the effects of feeding Bt MON810 maize to pigs for 110 days on the intestinal microbiota.

**Methodology/Principal Findings:**

Forty male pigs (∼40 days old) were blocked by weight and litter ancestry and assigned to one of four treatments; 1) Isogenic maize-based diet for 110 days (**Isogenic**); 2) Bt maize-based diet (MON810) for 110 days (**Bt**); 3) Isogenic maize-based diet for 30 days followed by a Bt maize-based diet for 80 days (**Isogenic/Bt**); 4) Bt maize-based diet for 30 days followed by an isogenic maize-based diet for 80 days (**Bt/Isogenic**). *Enterobacteriaceae*, *Lactobacillus* and total anaerobes were enumerated in the feces using culture-based methods on days 0, 30, 60 and 100 of the study and in ileal and cecal digesta on day 110. No differences were found between treatments for any of these counts at any time point. The relative abundance of cecal bacteria was also determined using high-throughput 16 S rRNA gene sequencing. No differences were observed in any bacterial taxa between treatments, with the exception of the genus *Holdemania* which was more abundant in the cecum of pigs fed the isogenic/Bt treatment compared to pigs fed the Bt treatment (0.012 vs 0.003%; *P*≤0.05).

**Conclusions/Significance:**

Feeding pigs a Bt maize-based diet for 110 days did not affect counts of any of the culturable bacteria enumerated in the feces, ileum or cecum. Neither did it influence the composition of the cecal microbiota, with the exception of a minor increase in the genus *Holdemania*. As the role of *Holdemania* in the intestine is still under investigation and no health abnormalities were observed, this change is not likely to be of clinical significance. These results indicate that feeding Bt maize to pigs in the context of its influence on the porcine intestinal microbiota is safe.

## Introduction

As the relationship between dietary habits and intestinal microbiota is becoming clearer with respect to a variety of illnesses, such as diabetes, obesity and inflammatory bowel disease [1], [2], [3], [4], consumers are becoming increasingly aware of the impact of certain foodstuffs on the intestinal microbiota [5]. This comes at a time when the controversy surrounding genetically modified (GM) food and animal feed is far from being resolved [6], [7], [8], thus fuelling potential consumer concerns about safety in relation to the intestinal microbiota.

Bt maize expressing the insecticidal Cry1Ab protein has been thoroughly tested during pre-market risk assessment and has been approved for inclusion in food and feed [9]. However, studies investigating its effects on animal health and production have not provided a definitive answer as to its safety [10], [11].

As studies investigating the antimicrobial properties of the Cry1Ab protein *in-vitro* have yielded contradictory results [12], [13], *in-vivo* studies are required to clarify this issue. Several groups, including ours, have investigated the effect of Bt maize on the intestinal microbiota in short-term pig-feeding studies [14], cattle studies [15], [16] and long-term sheep studies [17]. However, short-term studies may fail to adequately address consumer concerns, which are mainly related to the safety of Bt maize following long-term consumption. Also, pigs are a more suitable model for humans than ruminants, both physiologically and anatomically, as well as in terms of composition of the intestinal microbiota [18]. Therefore, feeding studies in pigs are more likely to provide an accurate insight into the potential impact of Bt maize in humans.

As culture-based microbiological analysis is becoming increasingly outdated, novel, more powerful methods, such as high-throughput 16 S rRNA gene sequencing are becoming increasingly popular for analysis of the intestinal microbiota. Such technologies offer the potential to generate large amounts of data [19], [20] and may prove to be a useful tool for testing the safety of Bt maize.

Therefore, the objective of this study was to investigate the effect of long-term (110 days) feeding of Bt maize to pigs on the intestinal microbiota using both culture-dependent and -independent methods. In addition, we investigated the effect of changing diets following 30 days of feeding to assess the possibility of a carry-over effect as a result of exposure to Bt maize early in life.

## Results

At approximately day 70 of the study, two pigs from the isogenic treatment and one from the Bt treatment displayed symptoms of diarrhoea and were treated with injectable Enrofloxacin (2.5 mg/kg body weight) for three consecutive days. As a result, data from these pigs were not included in the analysis.

### Maize and Diets

Both the proximate composition and the amino acid content of the isogenic and the Bt diets were similar [21]. The Bt maize used in this study was previously found to have a higher starch and sucrose content and a lower content of enzyme resistant starch than the isogenic maize [22]; however the amino acid profile and proximate composition were within the normal range for maize [22], [23], [24]. The concentrations of mycotoxins and pesticides in the isogenic and Bt maize were previously reported to be below the maximum allowable limits for animal feedstuffs [22].

### Culture-based Investigation of the Effects of Feeding Bt Maize on the Intestinal Microbiota of Pigs

No significant differences were found between the four dietary treatments for fecal bacterial counts of *Enterobacteriaceae*, *Lactobacillus* or total anaerobes on day 30, 60 or 100 (Table 1). *Enterobacteriaceae* counts increased from day 30 to 100 (*P*<0.05) and total anaerobe counts decreased over time for all treatments (*P*<0.05). Similar to fecal counts, ileal and cecal counts of *Enterobacteriaceae, Lactobacillus* and total anaerobes did not differ between treatments (Table 2).

**Table 1:** Effect of feeding isogenic or Bt maize-based diets to pigs from 12 days post-weaning for 110 days on fecal bacterial counts^1^.

Day	Treatments				Mean	SEM	*P*-value^6^		
	Isogenic^2^	Bt^3^	Isogenic/Bt^4^	Bt/isogenic^5^			Treatment	Time	Treatment×Time
*Enterobacteriaceae*									
30	5.47	6.08	5.45	5.40	5.60	0.192	0.57		
60	6.36	6.74	6.40	6.60	6.53	0.205	0.94		
100	7.50	7.14	7.29	7.15	7.27	0.202	0.95		
Mean	6.44	6.65	6.38	6.39		0.417	0.92	<0.0001	0.61
*Lactobacillus*									
30	8.47	8.71	8.75	8.61	8.63	0.153	0.94		
60	7.97	8.63	8.52	8.28	8.35	0.163	0.58		
100	8.25	8.45	8.54	8.50	8.43	0.163	0.95		
Mean	8.23	8.60	8.60	8.47		0.314	0.79	0.27	0.97
Total anaerobes									
30	9.79	9.86	9.96	9.86	9.87	0.097	0.95		
60	9.15	9.66	9.73	9.50	9.51	0.106	0.33		
100	9.18	9.49	9.27	9.47	9.35	0.103	0.77		
Mean	9.37	9.67	9.65	9.61		0.191	0.68	0.0002	0.71

1 Bacterial counts are presented as means of log_10_ CFU g^−1^ wet weight.

2 Isogenic - isogenic parent line maize-based diet for 110 days (n = 8 pigs/treatment).

3 Bt - Bt maize-based diet for 110 days (n = 9 pigs/treatment).

4 Isogenic/Bt - isogenic maize-based diet for 30 days followed by a Bt maize-based diet for 80 days (n = 10 pigs/treatment).

5 Bt/isogenic - Bt maize-based diet for 30 days followed by a isogenic maize-based diet for 80 days (n = 10 pigs/treatment).

6 Computed using the *mixed* procedure in SAS.

**Table 2:** Effect of feeding isogenic or Bt maize-based diets to pigs from 12 days post-weaning for 110 days on cecal and ileal bacterial counts^1^.

	Treatments				SEM	*P*-value^6^
	Isogenic^2^	Bt^3^	Isogenic/Bt^4^	Bt/isogenic^5^		
*Enterobacteriaceae*						
Ileum	8.34	7.29	7.63	6.64	0.423	0.24
Cecum	7.96	7.21	7.34	7.04	0.292	0.22
*Lactobacillus*						
Ileum	6.11	6.08	6.96	5.91	0.500	0.21
Cecum	7.94	7.09	7.14	7.28	0.452	0.88
Total anaerobes						
Ileum	8.59	8.03	8.14	7.83	0.322	0.55
Cecum	9.13	9.22	8.91	9.02	0.143	0.33

1 Bacterial counts are presented as log_10_ CFU g^−1^ wet weight.

2 Isogenic - isogenic parent line maize-based diet for 110 days (n = 8 pigs/treatment).

3 Bt - Bt maize-based diet for 110 days (n = 9 pigs/treatment).

4 Isogenic/Bt - isogenic maize-based diet for 30 days followed by a Bt maize-based diet for 80 days (n = 10 pigs/treatment).

5 Bt/isogenic - Bt maize-based diet for 30 days followed by a isogenic maize-based diet for 80 days (n = 10 pigs/treatment).

6 Computed using a one-way ANOVA in SAS.

### Effects of Feeding Bt Maize on the Relative Abundance of the Cecal Microbiota of Pigs

Raw sequence data has been uploaded to http://www.ebi.ac.uk, database reference number ERP001333.

A total of 151,608 16 S rRNA reads (239 bp long) were generated from high-throughput sequencing corresponding to an average of 4,097 reads per cecal sample (ranging from 2,251 to 10,296 reads per sample). From this, 138,854 (91.6%) were assigned at the phylum level, 79,368 (52.4%) at the family level and 58,914 (38.9%) at the genus level. Rarefaction curves were similar between treatments (Figures S1, S2, S3 and S4). The Shannon diversity index (an unweighted measure of the number of species present in a community [25]) was similar across all four treatments (Table 3). Likewise, Good’s coverage and Chao 1 richness estimator were similar between treatments (Table 3). No clustering corresponding to a specific treatment group was observed following beta diversity analysis (Figure S5).

**Table 3:** Estimation of bacterial diversity at 97% similarity in the cecum of pigs fed isogenic or Bt maize-based diets^1,2^.

	Treatments			
	Isogenic^3^	Bt^4^	Isogenic/Bt^5^	Bt/isogenic^6^
Chao 1 richness estimation	1238	1390	1451	1388
Shannon diversity index	5.56	5.90	5.77	5.77
Good’s coverage	0.91	0.91	0.91	0.91

1 Estimates of diversity were computed using MOTHUR software.

2 Data presented as treatment means.

3 Isogenic - isogenic parent line maize-based diet was fed for 110 days (n = 8 pigs/treatment).

4 Bt - Bt MON810 maize-based diet was fed for 110 days (n = 9 pigs/treatment).

5 Isogenic/Bt - isogenic maize-based diet was fed for 30 days followed by a Bt MON810 maize-based diet for 80 days (n = 10 pigs/treatment).

6 Bt/isogenic - Bt MON810 maize-based diet was fed for 30 days followed by a isogenic maize-based diet for 80 days (n = 10 pigs/treatment).

**Figure 1:**
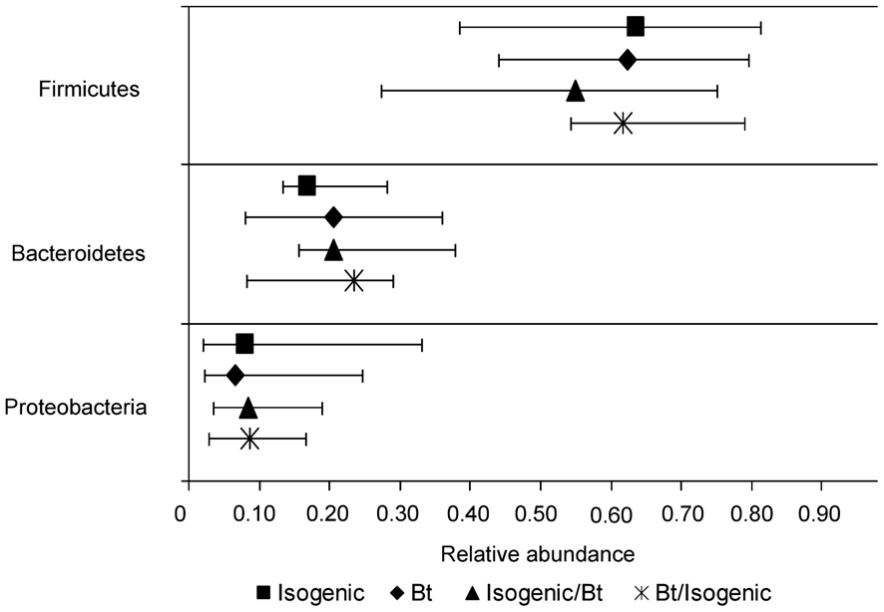
Effect of feeding Bt maize to pigs on relative abundance of major cecal bacterial phyla. Data presented as medians ±5^th^–95^th^ percentiles. A full outline of the relative abundance of all bacterial taxa in the porcine cecum is available in Table S1 and minor taxa which were statistically significant or showed tendencies towards significance are presented in Table 4. Isogenic - isogenic parent line maize-based diet was fed for 110 days (n = 8 pigs/treatment). Bt - Bt maize-based diet was fed for 110 days (n = 9 pigs/treatment). Isogenic/Bt - isogenic maize-based diet was fed for 30 days followed by Bt maize-based diet for 80 days (n = 10 pigs/treatment). Bt/isogenic - Bt maize-based diet was fed for 30 days followed by isogenic maize-based diet for 80 days (n = 10 pigs/treatment).

**Figure 2:**
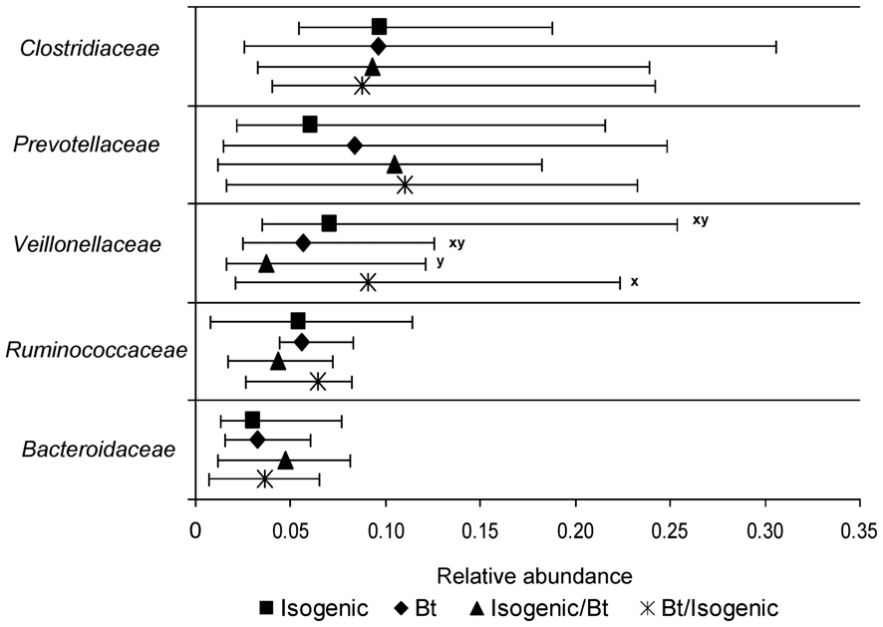
Effect of feeding Bt maize to pigs on relative abundance of major cecal bacterial families. Data presented as medians ±5^th^–95^th^ percentiles. A full outline of the relative abundance of all bacterial taxa in the porcine cecum is available in Table S1 and minor taxa which were statistically significant or showed tendencies towards significance are presented in Table 4. Isogenic - isogenic parent line maize-based diet was fed for 110 days (n = 8 pigs/treatment). Bt - Bt maize-based diet was fed for 110 days (n = 9 pigs/treatment). Isogenic/Bt - isogenic maize-based diet was fed for 30 days followed by Bt maize-based diet for 80 days (n = 10 pigs/treatment). Bt/isogenic - Bt maize-based diet was fed for 30 days followed by isogenic maize-based diet for 80 days (n = 10 pigs/treatment). ^x,y^Medians with different superscripts indicate a tendency towards statistical significance (0.05<*P*<0.10).

**Figure 3:**
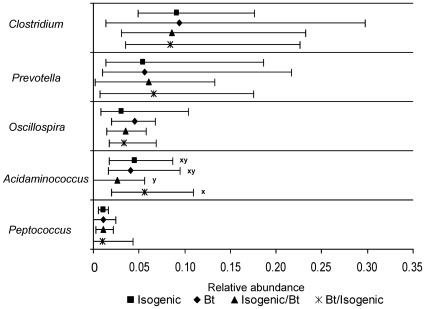
Effect of feeding Bt maize to pigs on relative abundance of major cecal bacterial genera. Data presented as medians ±5^th^–95^th^ percentiles. A full outline of the relative abundance of all bacterial taxa in the porcine cecum is available in Table S1 and minor taxa which were statistically significant or showed tendencies towards significance are presented in Table 4. Isogenic - isogenic parent line maize-based diet was fed for 110 days (n = 8 pigs/treatment). Bt - Bt maize-based diet was fed for 110 days (n = 9 pigs/treatment). Isogenic/Bt - isogenic maize-based diet was fed for 30 days followed by Bt maize-based diet for 80 days (n = 10 pigs/treatment). Bt/isogenic - Bt maize-based diet was fed for 30 days followed by isogenic maize-based diet for 80 days (n = 10 pigs/treatment). ^x,y^Medians with different superscripts indicate a tendency towards statistical significance (0.05<*P*<0.10).

A full outline of the relative abundance of all bacterial taxa in the porcine cecum is available in Table S1. Major bacterial taxa are presented in Figures 1, 2 and 3, while minor taxa which were statistically significant or showed tendencies towards significance are presented in Table 4. A total of 15 phyla were detected in the cecum of the 150 day old pigs with Firmicutes being the most abundant (61.5%) followed by Bacteroidetes (19.6%) and Proteobacteria (8.3%; Figure 1). Together, these three major phyla accounted for 89% of the porcine cecal bacteria, while the other 12 phyla made up the remaining 11% (Table S1). No difference between treatments was observed with respect to the relative abundance of bacterial phyla in the cecum.

**Table 4:** Effect of feeding isogenic or Bt maize-based diet to pigs from 12 days post weaning for 110 days on the relative abundance of cecal bacterial taxa in pigs^1^.

	Treatments					
	Isogenic^2^	Bt^3^	Isogenic/Bt^4^	Bt/isogenic^5^	5–95^th^ percentiles	*P*-value^6^
**Family**						
*Succinivibrionaceae*	0.012^x^	0.019^x^	0.003^y^	0.012^x^	0–0.14	0.08^†^
*Erysipelotrichaceae*	0.020^xy^	0.010^y^	0.022^x^	0.018^xy^	0.003–0.037	0.07*
**Genus**						
*Succinivibrio*	0.012^x^	0.019^x^	0.003^y^	0.012^x^	0–0.10	0.07^†^
*Eubacterium*	0^y^	0^y^	0.0017^xy^	0.0022^x^	0–0.008	0.09^†^
*Holdemania*	0.005^ab^	0.003^b^	0.012^a^	0.007^ab^	0–0.03	0.05^†^

1 Data reported as median values.

2 Isogenic - isogenic parent line maize-based diet for 110 days (n = 8 pigs/treatment).

3 Bt - Bt maize-based diet for 110 days (n = 9 pigs/treatment).

4 Isogenic/Bt - isogenic maize-based diet for 30 days followed by a Bt maize-based diet for 80 days (n = 10 pigs/treatment).

5 Bt/isogenic - Bt maize-based diet for 30 days followed by a isogenic maize-based diet for 80 days (n = 10 pigs/treatment).

6 Computed using a one-way ANOVA (*) or the Kruskal-Wallis non parametric test (†) in SAS.

Within each row, medians with different superscripts are different at ^a,b^ *P*≤0.05 or ^x,y^ *P*<0.10.

The 16 S rRNA reads from the cecum of 150 day old pigs were assigned to a total of 36 families. The porcine cecal microbiota was dominated by *Clostridiaceae* (9.6%), *Prevotellaceae* (9.1%), *Veillonellaceae* (6.2%), *Ruminococcaceae* (5.2%) and *Bacteroidaceae* (3.8%; Figure 2). No significant differences in relative abundance were detected between treatments for any of the bacterial families. However, although not reaching statistical significance, *Veillonellaceae* tended to be less abundant in the cecum of pigs fed the isogenic/Bt treatment compared to pigs fed the Bt/isogenic treatment (3.7 vs. 9%; *P* = 0.08; Figure 2) but was not different from the other two treatments. Also, *Succinivibrionaceae* tended to be lower in the cecum of pigs fed the isogenic/Bt treatment compared to pigs fed the isogenic, Bt and the isogenic/Bt treatments (*P* = 0.08; Table 4) but this difference was not statistically significant. In contrast, *Erysipelotrichaceae*, although not significantly different, tended to be more abundant in the cecum of pigs fed the isogenic/Bt treatment compared to pigs fed the Bt treatment (*P* = 0.07; Table 4) but was not different from the other two treatments. There was no effect of feeding Bt maize to pigs on any of the remaining families identified (Table S1).

Sequencing analysis identified 49 genera within the cecum of 150 day old pigs. Almost one quarter of the sequenced bacteria in the pig cecum (23.9%) were comprised of the genera *Clostridium* (9.1%), *Prevotella* (6.2%), *Oscillospira* (3.8%), *Acidaminococcus* (3.7%) and *Peptococcus* (1.1%; Figure 3). No significant differences were observed between treatments at the genus level, with the exception of *Holdemania.* This genus was more abundant in the cecum of pigs fed the isogenic/Bt treatment than in the cecum of pigs fed the Bt treatment (*P*≤0.05; Table 4) but was not different to pigs fed the isogenic or the Bt/isogenic treatments. Although statistical significance was not reached, there was a tendency for lower relative abundance of *Succinivibrio* in the cecum of pigs fed the isogenic/Bt treatment compared to all other treatments (*P* = 0.07; Table 4). *Acidaminococcus* also tended to be less abundant in the cecum of pigs fed the isogenic/Bt treatment compared to pigs fed the Bt/isogenic treatment (*P* = 0.09; Figure 3) but was not different from the isogenic or Bt treatments. *Eubacterium* were only detected in pigs fed the isogenic/Bt and Bt/isogenic treatments and not in those fed the Bt and isogenic treatments. Consequently, although not statistically significant, *Eubacterium* tended to have greater abundance in the cecum of pigs fed the Bt/isogenic treatment compared to pigs fed the isogenic and the Bt treatments (*P = *0.09; Table 4) but was not different from pigs fed the isogenic/Bt treatment.

## Discussion

When considering the impact of GM food and feed on the intestinal microbiota, horizontal transfer of the transgene to the microbiota is one of the major safety concerns [26]. However, gene transfer was not the focus of the present study and in fact, we have investigated it in a previous study (Buzoianu *et al.*, unpublished). The purpose of the present study was to investigate effects of the Bt maize on microbial community structure within the porcine intestinal tract. To our knowledge, this is the first pig feeding study to evaluate the effects of long-term exposure of Bt maize on intestinal microbial communities as assessed by high-throughput 16 S rRNA gene sequencing. A limited number of studies have investigated the effect of short-term feeding of Bt maize on porcine [14] or bovine intestinal microbiota using molecular techniques, such as 16 S rRNA gene sequencing [15] or real-time PCR [16]. However, these studies were conducted in weanling pigs fed Bt maize for only 31 days [14] or in mature cattle fed Bt maize for four weeks [15] or 11 days [16]. Such studies provide valuable insight into the effect of Bt maize on both an unstable microbial community in young animals or an established climax community in mature animals [15], [16]. However, evidence suggests that six weeks of exposure are required for the intestinal microbiota to adapt to feed structure [27], which highlights the limitations of short-term feeding studies. Other potential limitations relate to fears expressed by some consumers that health effects arising from exposure to GM crops may only become evident following long-term exposure [28]. In addition, differences in digestive physiology between humans and ruminants [29] make the latter less suitable as a human model. By conducting a long-term feeding study throughout the pig’s entire productive life we have increased the potential to detect discrete changes in intestinal microbiota that may not be obvious following short-term exposure.

The absence of an effect of Bt maize on fecal, ileal and cecal counts of *Enterobacteriaceae*, *Lactobacillus* and total anaerobes is in agreement with previous findings by our group following 31 days of Bt maize exposure in weanling pigs [14]. Similarly, Bt rice, expressing the Cry1Ab protein was found to have no impact on fecal coliforms, *Lactobacillus* or total anaerobes in rats following 90 days of consumption [30]. However, in contrast to our findings, the latter study found that coliform counts increased in the ileum and counts of bifidobacteria decreased in the duodenum of rats fed Bt rice.

Fecal *Lactobacillus* counts were found to be stable over time in the present study. This is in agreement with published literature which indicates that lactobacilli reach a stable community after the first week of life [27], [31], [32]. Likewise, the rise in *Enterobacteriaceae* counts over time reported in the present study is in agreement with previous studies which found lower counts during the first 30 days post-weaning [33] and higher counts as pigs mature [34]. Similar to published findings for pigs [35], [36], [37], fecal counts of total anaerobes remained high throughout the study.

Our findings also agree with previous data from our group [14] and others [38], [39], [40] in that the porcine cecum was dominated by the phyla Firmicutes, Bacteroidetes and Proteobacteria, although in previous studies the proportions differed depending on age and diet. Similarly, in humans, Firmicutes and Bacteroidetes comprise the “core” bacteria of the large intestine [41], [42] and have been used as biomarkers for the metabolic status of the host [43], [44]. In accordance with our previous research in 60 day-old pigs [14] and with results from Kim *et al*. [40] in 22 week-old pigs, *Clostridiaceae* and *Prevotellaceae* were the dominant families in the pig cecum in the present study. Similarly, Leser *et al.* [45] also found that *Clostridiaceae, Prevotellaceae* and *Bacteroides* dominated the digestive tract of pigs of different ages fed different diets. *Clostridia* and *Prevotellaceae* have also been recovered in high numbers from human intestinal and fecal samples [44], [46], [47], [48]. These similarities underline the value of the pig as a model for predicting the influence of Bt maize on the major taxa of the human intestinal microbiota.

The composition of the cecal microbiota was similar for the isogenic maize control treatment and the Bt maize treatment in the present study. Similarly, real-time PCR analysis revealed no effects of feeding Bt176 maize silage on any of six ruminal bacterial species in cows [16]. Another study also demonstrated that feeding Bt176 maize silage to cows for four weeks did not influence the composition of ruminal microbiota as assessed by 16 S rRNA gene sequencing [15]. Likewise, total ruminal amylolytic and cellulolytic bacterial populations, as well as protozoal numbers and composition and microbial metabolites did not differ between sheep fed Bt176 or non-GM maize for three years [17].

The only statistically significant difference that was observed within the cecal microbiota in the present study was that the genus *Holdemania* was more abundant in pigs fed the isogenic/Bt treatment compared to pigs fed the Bt treatment. This difference may be more related to the changing of maize source at a time when the intestinal microbiota is not yet fully established than to the nature of the change (i.e. from isogenic to Bt or vice versa). Although the presence of *Holdemania* at low relative abundance has been established in the porcine intestine [40], [45], the role of this genus in the intestine is not fully understood. In the present study, the difference in cecal *Holdemania* abundance was not associated with any effects on small intestinal weight or morphology [21]. Furthermore, none of the minor differences in blood biochemistry observed between treatments in these pigs [21] could be related to changes in the intestinal microbiota. Therefore, although statistically significant, the difference in cecal *Holdemania* abundance observed in the present study is not believed to be of biological significance or to have a major impact on pig health. The increased abundance in cecal *Holdemania* is believed to be related to the time at which maize source was changed rather than a response to feeding Bt maize *per se*.

In conclusion, no changes were observed within the cecal microbial community of healthy pigs following long-term exposure to Bt maize or following a cross-over between isogenic and Bt maize after 30 days of feeding, with the exception of *Holdemania*. The fact that no difference between the Bt and isogenic treatments was observed provides evidence that the intestinal microbiota are tolerant to Bt maize and substantiates our previous findings that Bt maize is safe for long-term consumption. Changing maize source following 30 days of feeding did not affect the intestinal microbiota with the exception of *Holdemania* which indicates the absence of a residual effect following Bt maize exposure early in life. Also, neither Bt maize nor changing maize source affected counts of fecal *Enterobacteriaceae*, *Lactobacillus* or total anaerobes at any time during the study. These findings indicate that Bt maize is well tolerated by the ‘normal’ intestinal microbiota of healthy pigs and fails to alter its composition, at least in the cecum, even after long-term exposure. However, as stress is known to affect the response to stimuli, future studies are needed to investigate potential effects of Bt maize in physiologically stressed animals.

## Materials and Methods

### Ethical Approval

The pig study complied with European legislation concerning minimum standards for pig protection (European Union Council Directive 91/630/EEC) and the protection of animals kept for farming purposes (European Union Council Directive 98/58/EC). Ethical approval was obtained from the ethics committees of Teagasc and Waterford Institute of Technology. An experimental license (number B100/4147) was obtained from the Irish Department of Health and Children.

### Animals and Experimental Design

Forty crossbred (Large White×Landrace) entire male pigs were weaned at ∼ 28 days of age and allowed a 12 day adaptation period. During this adaptation period, pigs were provided with *ad libitum* access to a non-GM starter diet. Pigs were then blocked by weight and ancestry and, within block, randomly assigned to one of four treatments at ∼40 days of age (n = 10 pigs/treatment); 1) isogenic maize-based diet (isogenic parent line; Pioneer *PR34N43*) for 110 days (**isogenic**); 2) Bt maize-based diet (Bt; Pioneer *PR34N44*; event MON810) for 110 days (**Bt**); 3) Isogenic maize-based diet for 30 days followed by Bt maize-based diet for 80 days (**isogenic/Bt**); and 4) Bt maize-based diet for 30 days followed by isogenic maize-based diet for 80 days (**Bt/isogenic**). The duration of the study was 110 days. Pigs were individually housed in identical pens in similar climatically controlled rooms and were allowed *ad libitum* access to water and feed. For the duration of the study, all dietary treatments were equally represented in each room to remove any variation due to environmental factors. Pigs showing signs of ill health were treated as appropriate and all veterinary treatments were recorded.

### Maize and Diets

In accordance with established guidelines [23], [26], [49], the maize lines used in the present study were MON810 maize and its closest comparator, the isogenic maize from which it was derived. Furthermore, to ensure similar growing conditions, Bt MON810 maize and its isogenic counterpart (PR34N44 and PR34N43, respectively; Pioneer Hi-Bred, Seville, Spain) were grown in neighbouring plots in Valtierra, Navarra, Spain by independent farmers. The Bt and isogenic control maize were purchased by the authors from the tillage farmers for use in this animal study. Samples from the isogenic and Bt maize were tested for the presence of the *cry1Ab* transgene and for the presence of pesticide contaminants and mycotoxins as described by Walsh *et al*. [22]. Proximate composition and amino acid content of the maize and diets, as well as carbohydrate composition of the maize, were also determined as described by Walsh *et al*. [22].

All diets were formulated to meet or exceed the National Research Council requirements for pigs [50] and were manufactured as outlined by Walsh *et al*. [22]. The isogenic and Bt diets were formulated with identical maize inclusion rates. As a precautionary measure a mycotoxin binder was included in all diets used in the study (Mycosorb™, Alltech, Dunboyne, Co. Meath, Ireland). A succession of diets was fed to pigs according to their age group as follows: link diets from day 0 to 30, weaner diets from day 31 to 60, finisher 1 diets from day 61 to 100 and finisher 2 diets from day 101 to 110. The composition of the diets has previously been reported by Buzoianu *et al*. [21].

### Fecal and Cecal and Ileal Digesta Sampling and Microbiological Analysis

Fecal samples were collected in sterile containers by rectal stimulation from 10 pigs/treatment on days 0, 30, 60 and 100. Digesta samples from the cecum (terminal tip of the cecum) and ileum (15 cm before the ileo-cecal junction) were obtained following euthanasia on day 110 when pigs were ∼150 days of age. The last meal was provided 3 hours before euthanasia. Digesta samples were removed aseptically, placed in sterile plastic containers and stored at 4°C in sealed anaerobic jars containing Anaerocult™ A gas packs (Merck, Darmstadt, Germany) until analysis (within 12 hours). Enumeration of *Lactobacillus* and *Enterobacteriaceae* from individual fecal samples and ileal and cecal digesta was performed as described by Gardiner *et al.* [33]. To inhibit growth of yeasts and moulds, nystatin (Sigma Aldrich Ireland Ltd., Wicklow, Ireland) was added to the *Lactobacillus* selective medium at a concentration of 50 units/mL. Total anaerobic bacteria from individual fecal, ileal and cecal samples were enumerated as previously described by Rea *et al*. [51].

### DNA Extraction and PCR

The QIAamp DNA Stool kit (Qiagen, Crawley, West Sussex, UK) was used to extract total DNA from individual cecal digesta samples, according to the manufacturer’s instructions with some modifications. To increase DNA yield an additional bead beating step was included [52] and the initial lysis temperature was increased from 70 to 90°C. For PCR, forward and reverse primers targeting the V4 region of the bacterial 16 S rRNA gene were used, as previously described by Murphy *et al*. [53]. These primers are predicted to bind to 96.4% of all 16 S rRNA genes [53]. To allow detection of individual amplicons from samples that were pooled at the sequencing stage, unique molecular identifiers were incorporated into the forward primer (Table S2) [53].

Each PCR reaction contained 2 µL of template DNA, 200 nM of forward primer, and 50 nM of each of the four reverse primers, 25 µL of Biomix Red (Bioline, London, UK) and 21 µL of sterile double distilled water. Each set of PCR reactions contained a negative control, in which template DNA was replaced with sterile double distilled water and a positive control containing previously amplified cecal bacterial DNA. The PCR cycle started with denaturation at 94°C for 2 minutes, followed by 35 cycles of denaturation (94°C for 1 minute), annealing (52°C for 1 minute) and elongation (72°C for 1 minute). A final elongation step was performed at 72°C for 2 minutes. Amplicons were detected by UV visualization following electrophoresis in 1.5% agarose gels containing 0.3 ng/µL ethidium bromide. PCR products were purified using the High Pure PCR product purification kit (Roche Applied Science, Mannheim, Germany). DNA was quantified using a NanoDrop 3300 spectrophotometer (Fisher Scientific, DE, USA) following staining using the Quant-it Pico Green dsDNA kit (Invitrogen Ltd. Paisley, UK).

### 16S rRNA Gene Sequencing

Sequencing was performed on a 454 Genome Sequencer FLX platform (Roche Diagnostics Ltd., Burgess Hill, West Sussex), according to manufacturer’s instructions. De-noising, sequence length cut-off, quality score cut-off, checking of sequencing reads and assignment to NCBI taxonomies were performed as previously described by Murphy *et al.* [53]. Principal coordinate analysis was performed using the QIIME software tool [54]. Population indices, such as Chao1 richness estimation, Shannon diversity and Good’s coverage were computed at the genus level using MOTHUR software [55].

The number of reads assigned to each cecal bacterial taxonomical rank was divided by the total number of reads assigned to the highest rank (phylum) to obtain the relative abundance values. Therefore, relative abundance is presented as a ratio, with values ranging from 0 (0%) to 1 (100%).

### Statistical Analysis

To ensure normality, bacterial counts and relative abundance values were log-transformed to the base 10. Fecal bacterial counts were analysed with sampling day as a repeated measure using the MIXED procedure in SAS version 9.1.3 [56] with day 0 values as a covariate in the model. Fixed effects included treatment and sampling day, while block was included as a random effect in the model. The *slice* option was used to determine significance for simple main effects. Ileal and cecal bacterial counts, as well as the relative abundance data which were normally distributed were analysed as a complete randomized block design using the GLM procedure in SAS. Data which were not normally distributed following log-transformation were analysed using the non-parametric Kruskal-Wallis test within the NPAR1WAY procedure in SAS. In this case, data were presented as treatment median values and the 5–95^th^ percentiles. Statistical significance was considered for *P*≤0.05 and tendencies were reported up to *P* = 0.10. Means separation was performed using the Tukey-Kramer post-hoc adjustment for normally distributed data. For data that were analysed using the Kruskal-Wallis non-parametric test, significance between treatments was determined by using the GLM procedure in SAS on the Wilcoxon signed ranks and means separation was performed using the Tukey-Kramer post-hoc adjustment. For all analyses the individual pig was the experimental unit.

## Supporting Information

Figure S1 **Bacterial alpha diversity at 97% similarity in the cecum of ∼150 day-old pigs.** Pigs were fed an isogenic maize-based diet for 110 days. OTU - operational taxonomical unit.
(TIF)

Figure S2 **Bacterial alpha diversity at 97% similarity in the cecum of ∼150 day-old pigs.** Pigs were fed a Bt maize-based diet for 110 days. OTU - operational taxonomical unit.
(TIF)

Figure S3 **Bacterial alpha diversity at 97% similarity in the cecum of ∼150 day-old pigs.** Pigs were fed an isogenic maize-based diet for 30 days followed by a Bt maize-based diet for 80 days. OTU - operational taxonomical unit.
(TIF)

Figure S4 **Bacterial alpha diversity at 97% similarity in the cecum of ∼150 day-old pigs.** Pigs were fed a Bt maize-based diet for 30 days followed by an isogenic maize-based diet for 80 days. OTU - operational taxonomical unit.
(TIF)

Figure S5 **Unweighted bacterial beta diversity in the cecum of ∼150 day-old pigs.** Unweighted beta diversity was computed using QIIME software. Blue - isogenic maize-based diet was fed for 110 days. Green - Bt maize-based diet was fed for 110 days Red - isogenic maize-based diet was fed for 30 days followed by a Bt maize-based diet for 80 days. Light blue - Bt maize-based diet was fed for 30 days followed by a isogenic maize-based diet for 80 days.
(TIF)

Table S1 **Bacterial taxa detected in the cecum of ∼150 day-old pigs^1^.** ^1^Relative abundance presented as median values. Zero values correspond to taxa which were detected in a low number of samples per treatment with an abundance of <0.001. ^2^Isogenic - isogenic parent line maize-based diet for 110 days (n = 8 pigs/treatment). ^3^Bt - Bt maize-based diet for 110 days (n = 9 pigs/treatment). ^4^Isogenic/Bt - isogenic maize-based diet for 30 days followed by a Bt maize-based diet for 80 days (n = 10 pigs/treatment). ^5^Bt/isogenic - Bt maize-based diet for 30 days followed by a isogenic maize-based diet for 80 days (n = 10 pigs/treatment).
(DOC)

Table S2 **Individual molecular identifiers used for PCR amplification of the 16 S rRNA gene fragments from porcine cecal samples.** ^1^Removed from the analysis following antibiotic treatment. ^2^Isogenic - isogenic parent line maize-based diet for 110 days. ^3^Bt - Bt maize-based diet for 110 days. ^4^Isogenic/Bt - isogenic maize-based diet for 30 days followed by a Bt maize-based diet for 80 days. ^5^Bt/isogenic - Bt maize-based diet for 30 days followed by an isogenic maize-based diet for 80 days.
(DOC)
