# Supplementary figures and images for: The Effect of Feeding Bt MON810 Maize to Pigs for 110 Days on Intestinal Microbiota
Source: PLoS One. 2012 May 4;7(5):e33668. doi: 10.1371/journal.pone.0033668 (PMC3344822; doi:10.1371/journal.pone.0033668)

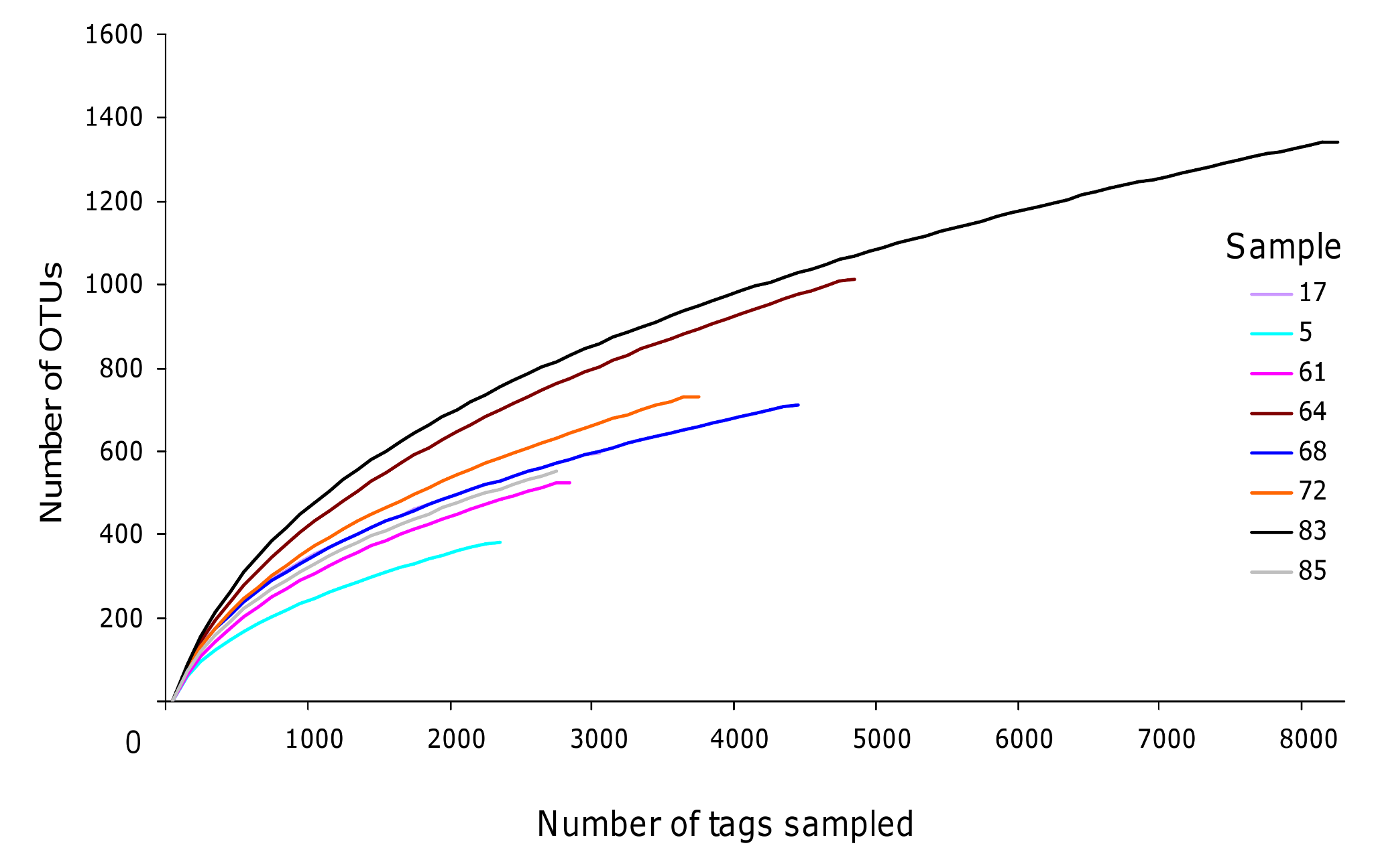

Supplement: Figure S1 — Bacterial alpha diversity at 97% similarity in the cecum of ∼150 day-old pigs. Pigs were fed an isogenic maize-based diet for 110 days. OTU - operational taxonomical unit. (TIF) [file pone.0033668.s001.tif]

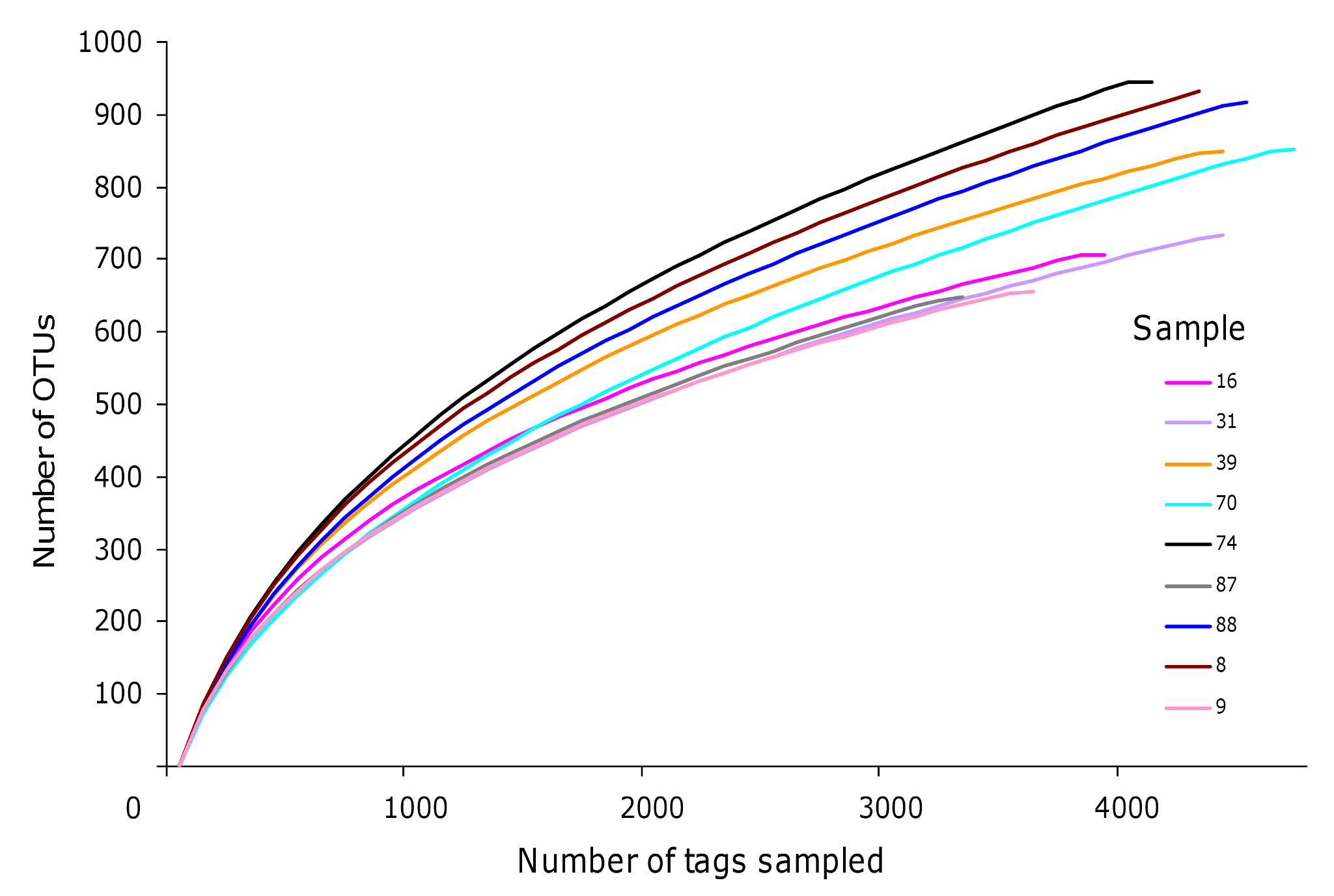

Supplement: Figure S2 — Bacterial alpha diversity at 97% similarity in the cecum of ∼150 day-old pigs. Pigs were fed a Bt maize-based diet for 110 days. OTU - operational taxonomical unit. (TIF) [file pone.0033668.s002.tif]

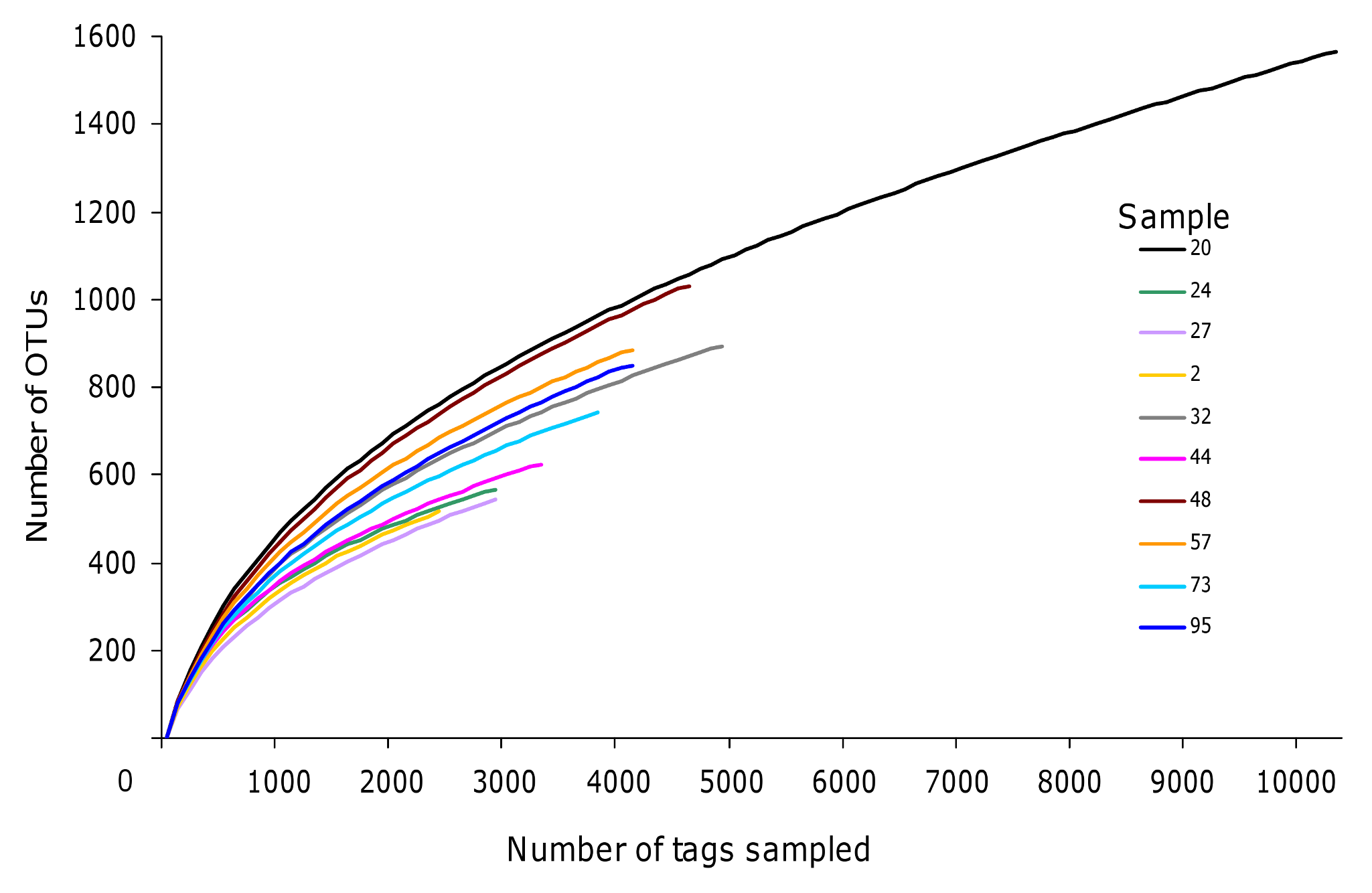

Supplement: Figure S3 — Bacterial alpha diversity at 97% similarity in the cecum of ∼150 day-old pigs. Pigs were fed an isogenic maize-based diet for 30 days followed by a Bt maize-based diet for 80 days. OTU - operational taxonomical unit. (TIF) [file pone.0033668.s003.tif]

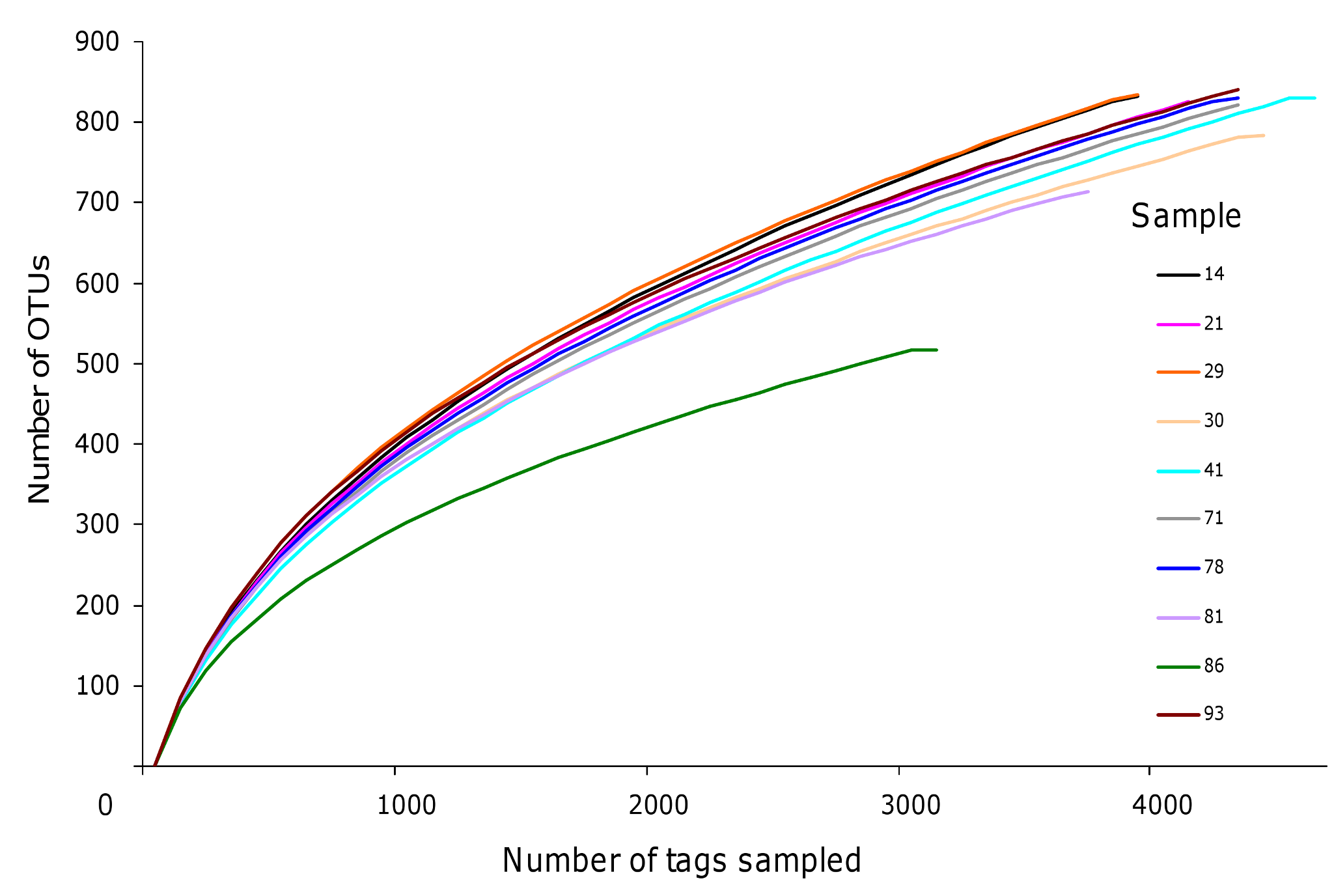

Supplement: Figure S4 — Bacterial alpha diversity at 97% similarity in the cecum of ∼150 day-old pigs. Pigs were fed a Bt maize-based diet for 30 days followed by an isogenic maize-based diet for 80 days. OTU - operational taxonomical unit. (TIF) [file pone.0033668.s004.tif]

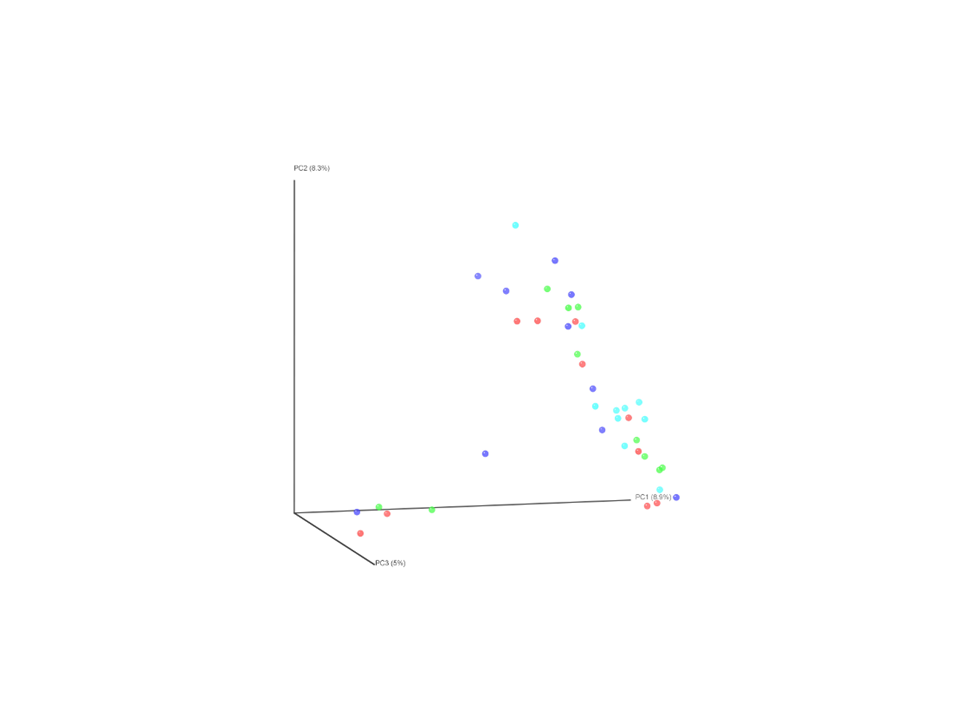

Supplement: Figure S5 — Unweighted bacterial beta diversity in the cecum of ∼150 day-old pigs. Unweighted beta diversity was computed using QIIME software. Blue - isogenic maize-based diet was fed for 110 days. Green - Bt maize-based diet was fed for 110 days Red - isogenic maize-based diet was fed for 30 days followed by a Bt maize-based diet for 80 days. Light blue - Bt maize-based diet was fed for 30 days followed by a isogenic maize-based diet for 80 days. (TIF) [file pone.0033668.s005.tif]
