# Supplementary material for: The Effect of Feeding Bt MON810 Maize to Pigs for 110 Days on Intestinal Microbiota
Source: PLoS One. 2012 May 4;7(5):e33668. doi: 10.1371/journal.pone.0033668 (PMC3344822; doi:10.1371/journal.pone.0033668)
Supplement: Table S1 — Bacterial taxa detected in the cecum of ∼150 day-old pigs1. 1Relative abundance presented as median values. Zero values correspond to taxa which were detected in a low number of samples per treatment with an abundance of <0.001. 2Isogenic - isogenic parent line maize-based diet for 110 days (n = 8 pigs/treatment). 3Bt - Bt maize-based diet for 110 days (n = 9 pigs/treatment). 4Isogenic/Bt - isogenic maize-based diet for 30 days followed by a Bt maize-based diet for 80 days (n = 10 pigs/treatment). 5Bt/isogenic - Bt maize-based diet for 30 days followed by a isogenic maize-based diet for 80 days (n = 10 pigs/treatment). (DOC) [file pone.0033668.s006.doc]

| **Phylum** | **Isogenic^2^** | **Bt^3^** | **Isogenic/ Bt^4^** | **Bt/Isogenic^5^** |
| --- | --- | --- | --- | --- |
| Firmicutes | 0.636 | 0.624 | 0.549 | 0.618 |
| Bacteroidetes | 0.169 | 0.207 | 0.206 | 0.234 |
| Proteobacteria | 0.080 | 0.066 | 0.084 | 0.086 |
| Spirochaetes | 0.011 | 0.008 | 0.006 | 0.012 |
| Verrucomicrobia | 0.000 | 0.007 | 0.017 | 0.006 |
| Planctomycetes | 0.000 | 0.002 | 0.001 | 0.003 |
| Lentisphaerae | 0.000 | 0.001 | 0.000 | 0.002 |
| Fusobacteria | 0.000 | 0.000 | 0.000 | 0.001 |
| Cyanobacteria | 0.000 | 0.000 | 0.000 | 0.000 |
| Deferribacteres | 0.000 | 0.000 | 0.000 | 0.000 |
| Fibrobacteres | 0.000 | 0.000 | 0.000 | 0.000 |
| Actinobacteria | 0.000 | 0.000 | 0.000 | 0.000 |
| Tenericutes | 0.000 | 0.000 | 0.000 | 0.000 |
| Chlamydiae | 0.000 | 0.000 | 0.000 | 0.000 |
| Elusimicrobia | 0.000 | 0.000 | 0.000 | 0.000 |
| **Family** |  |  |  |  |
| *Clostridiaceae* | 0.097 | 0.096 | 0.093 | 0.088 |
| *Prevotellaceae* | 0.060 | 0.084 | 0.104 | 0.111 |
| *Veillonellaceae* | 0.070 | 0.057 | 0.037 | 0.090 |
| *Ruminococcaceae* | 0.054 | 0.056 | 0.043 | 0.064 |
| *Bacteroidaceae* | 0.030 | 0.033 | 0.048 | 0.036 |
| *Erysipelotrichaceae* | 0.021 | 0.010 | 0.022 | 0.018 |
| *Desulfovibrionaceae* | 0.004 | 0.012 | 0.022 | 0.013 |
| *Peptococcaceae* | 0.011 | 0.007 | 0.010 | 0.010 |
| *Succinivibrionaceae* | 0.012 | 0.019 | 0.003 | 0.012 |
| *Spirochaetaceae* | 0.011 | 0.008 | 0.004 | 0.012 |
| *Lachnospiraceae* | 0.003 | 0.007 | 0.005 | 0.005 |
| *Enterobacteriaceae* | 0.009 | 0.006 | 0.004 | 0.005 |
| *Alcaligenaceae* | 0.005 | 0.004 | 0.004 | 0.005 |
| *Eubacteriaceae* | 0.003 | 0.004 | 0.004 | 0.005 |
| *Streptococcaceae* | 0.004 | 0.004 | 0.005 | 0.004 |
| *Lactobacillaceae* | 0.000 | 0.003 | 0.005 | 0.003 |
| *Verrucomicrobia subdivision 5* | 0.000 | 0.007 | 0.001 | 0.006 |
| *Rhodospirillaceae* | 0.000 | 0.000 | 0.000 | 0.000 |
| *Acetobacteraceae* | 0.000 | 0.000 | 0.000 | 0.000 |
| *Rickettsiaceae* | 0.000 | 0.000 | 0.000 | 0.000 |
| *Pasteurellaceae* | 0.000 | 0.000 | 0.001 | 0.000 |
| *Helicobacteraceae* | 0.000 | 0.001 | 0.000 | 0.001 |
| *Campylobacteraceae* | 0.000 | 0.000 | 0.000 | 0.000 |
| *Oxalobacteraceae* | 0.000 | 0.000 | 0.000 | 0.000 |
| *Rhodocyclaceae* | 0.000 | 0.000 | 0.000 | 0.000 |
| *Porphyromonadaceae* | 0.000 | 0.000 | 0.000 | 0.000 |
| *Clostridiales Family XIII Incertae Sedis* | 0.000 | 0.000 | 0.000 | 0.000 |
| *Fusobacteriaceae* | 0.000 | 0.000 | 0.000 | 0.000 |
| *Fibrobacteraceae* | 0.000 | 0.000 | 0.000 | 0.000 |
| *Bifidobacteriaceae* | 0.000 | 0.000 | 0.000 | 0.000 |
| *Coriobacteriaceae* | 0.000 | 0.000 | 0.000 | 0.000 |
| *Verrucomicrobia* | 0.000 | 0.000 | 0.000 | 0.000 |
| *Anaeroplasmataceae* | 0.000 | 0.000 | 0.000 | 0.000 |
| *Mycoplasmataceae* | 0.000 | 0.000 | 0.000 | 0.000 |
| *Chlamydiaceae* | 0.000 | 0.000 | 0.000 | 0.000 |
| *Victivallaceae* | 0.000 | 0.000 | 0.000 | 0.000 |
| **Genus** |  |  |  |  |
| *Clostridium* | 0.091 | 0.094 | 0.086 | 0.084 |
| *Prevotella* | 0.054 | 0.056 | 0.060 | 0.066 |
| *Oscillospira* | 0.030 | 0.045 | 0.035 | 0.034 |
| *Acidaminococcus* | 0.046 | 0.041 | 0.026 | 0.056 |
| *Peptococcus* | 0.011 | 0.011 | 0.010 | 0.010 |
| *Desulfovibrio* | 0.004 | 0.010 | 0.015 | 0.011 |
| *Succinivibrio* | 0.012 | 0.019 | 0.003 | 0.012 |
| *Holdemania* | 0.005 | 0.003 | 0.012 | 0.007 |
| *Treponema* | 0.010 | 0.008 | 0.004 | 0.006 |
| *Faecalibacterium* | 0.007 | 0.007 | 0.004 | 0.006 |
| *Sutterella* | 0.005 | 0.004 | 0.004 | 0.005 |
| *Turicibacter* | 0.004 | 0.003 | 0.003 | 0.006 |
| *Streptococcus* | 0.003 | 0.004 | 0.003 | 0.004 |
| *Ruminococcus* | 0.002 | 0.005 | 0.001 | 0.004 |
| *Lactobacillus* | 0.000 | 0.003 | 0.005 | 0.003 |
| *Eubacterium* | 0.000 | 0.000 | 0.002 | 0.002 |
| *Bacteroides* | 0.001 | 0.002 | 0.008 | 0.001 |
| *Rickettsia* | 0.000 | 0.000 | 0.000 | 0.000 |
| *Anaerobiospirillum* | 0.000 | 0.000 | 0.000 | 0.000 |
| *Actinobacillus* | 0.000 | 0.000 | 0.000 | 0.000 |
| *Helicobacter* | 0.000 | 0.001 | 0.000 | 0.001 |
| *Campylobacter* | 0.000 | 0.000 | 0.000 | 0.000 |
| *Spirochaeta* | 0.000 | 0.000 | 0.000 | 0.000 |
| *Odoribacter* | 0.000 | 0.000 | 0.000 | 0.000 |
| *Lactococcus* | 0.000 | 0.000 | 0.000 | 0.000 |
| *Acetitomaculum* | 0.000 | 0.000 | 0.000 | 0.000 |
| *Butyrivibrio* | 0.000 | 0.000 | 0.000 | 0.000 |
| *Lachnospira* | 0.000 | 0.000 | 0.000 | 0.000 |
| *Dorea* | 0.000 | 0.000 | 0.000 | 0.000 |
| *Coprococcus* | 0.000 | 0.000 | 0.000 | 0.002 |
| *Megasphaera* | 0.000 | 0.003 | 0.000 | 0.001 |
| *Sarcina* | 0.000 | 0.000 | 0.000 | 0.000 |
| *Veillonella* | 0.000 | 0.000 | 0.000 | 0.000 |
| *Mitsuokella* | 0.000 | 0.000 | 0.000 | 0.000 |
| *Blautia* | 0.000 | 0.000 | 0.000 | 0.000 |
| *Mogibacterium* | 0.000 | 0.000 | 0.000 | 0.000 |
| *Anaerofilum* | 0.000 | 0.000 | 0.000 | 0.000 |
| *Catenibacterium* | 0.000 | 0.000 | 0.000 | 0.002 |
| *Fibrobacter* | 0.000 | 0.000 | 0.000 | 0.000 |
| *Bifidobacterium* | 0.000 | 0.000 | 0.000 | 0.000 |
| *Spirulina* | 0.000 | 0.000 | 0.000 | 0.000 |
| *Olsenella* | 0.000 | 0.000 | 0.000 | 0.000 |
| *Anaeroplasma* | 0.000 | 0.000 | 0.000 | 0.000 |
| *Mycoplasma* | 0.000 | 0.000 | 0.000 | 0.000 |
| *Fusobacterium* | 0.000 | 0.000 | 0.000 | 0.000 |
| *Denitrobacterium* | 0.000 | 0.000 | 0.000 | 0.000 |
| *Chlamydia* | 0.000 | 0.000 | 0.000 | 0.000 |
| *Synechococcus* | 0.000 | 0.000 | 0.000 | 0.000 |
| *Victivallis* | 0.000 | 0.000 | 0.000 | 0.000 |
