# Supplementary material for: The Effect of Feeding Bt MON810 Maize to Pigs for 110 Days on Intestinal Microbiota
Source: PLoS One. 2012 May 4;7(5):e33668. doi: 10.1371/journal.pone.0033668 (PMC3344822; doi:10.1371/journal.pone.0033668)
Supplement: Table S2 — Individual molecular identifiers used for PCR amplification of the 16 S rRNA gene fragments from porcine cecal samples. 1Removed from the analysis following antibiotic treatment. 2Isogenic - isogenic parent line maize-based diet for 110 days. 3Bt - Bt maize-based diet for 110 days. 4Isogenic/Bt - isogenic maize-based diet for 30 days followed by a Bt maize-based diet for 80 days. 5Bt/isogenic - Bt maize-based diet for 30 days followed by an isogenic maize-based diet for 80 days. (DOC) [file pone.0033668.s007.doc]

| **Pig** | **Treatment** | **Molecular identifier** |
| --- | --- | --- |
| 52^1^ | Isogenic^2^ | AGAGAGAG |
| 64 | isogenic | AGAGCAGC |
| 68 | isogenic | AGATCATC |
| 17 | isogenic | AGATGAGC |
| 5 | isogenic | AGATGCTC |
| 83 | isogenic | ATCAGCTG |
| 85 | isogenic | ATCTCATC |
| 1^1^ | isogenic | ATCTCTGC |
| 61 | isogenic | ATCTGATG |
| 72 | isogenic | ATCTGCTC |
| 47^1^ | Bt^3^ | AGCAGAGC |
| 8 | Bt | AGCAGCAG |
| 74 | Bt | AGCATCTG |
| 16 | Bt | AGCTCATG |
| 9 | Bt | AGCTGCTG |
| 39 | Bt | ATGAGAGC |
| 87 | Bt | ATGAGCAG |
| 31 | Bt | ATGCATGC |
| 88 | Bt | ATGCTCAG |
| 70 | Bt | ATGCTCTC |
| 48 | isogenic/Bt^4^ | AGCAGATG |
| 24 | isogenic/Bt | AGCAGCTC |
| 2 | isogenic/Bt | AGCATGAG |
| 44 | isogenic/Bt | AGCTCAGC |
| 95 | isogenic/Bt | AGCTGATC |
| 73 | isogenic/Bt | ATGAGATG |
| 27 | isogenic/Bt | ATGAGCTC |
| 20 | isogenic/Bt | ATGATCTG |
| 57 | isogenic/Bt | ATGATGAG |
| 32 | isogenic/Bt | ATGCAGAG |
| 29 | Bt/isogenic^5^ | AGAGATGC |
| 30 | Bt/isogenic | AGAGCATG |
| 81 | Bt/isogenic | AGATCTGC |
| 86 | Bt/isogenic | AGATGATG |
| 21 | Bt/isogenic | AGATGCAG |
| 78 | Bt/isogenic | ATCAGATC |
| 93 | Bt/isogenic | ATCATCAG |
| 14 | Bt/isogenic | ATCATCTC |
| 41 | Bt/isogenic | ATCTGAGC |
| 71 | Bt/isogenic | ATCTGCAG |
